# Sexual orientation differences in female sexual dysfunction in a large cohort of nurses in older adulthood

**DOI:** 10.1093/jsxmed/qdag266

**Published:** 2026-08-27

**Authors:** Tabor Hoatson, Julia C Bond, Colleen A Reynolds, Julia L Marcus, Brittany M Charlton

**Affiliations:** Department of Epidemiology, Harvard T.H. Chan School of Public Health, Boston, MA, 02115, United States; Department of Epidemiology, Boston University School of Public Health, Boston, MA, 02118, United States; Erasmus MC, University Medical Center Rotterdam, Public Health, Rotterdam, the Netherlands; Erasmus MC, University Medical Center Rotterdam, Epidemiology, Rotterdam, 3015 GD, the Netherlands; Department of Population Medicine, Harvard Pilgrim Health Care Institute, Boston, MA, 02115, United States; Department of Population Medicine, Department of Population Medicine, Harvard Medical School, Boston, MA, 02115, United States; Department of Epidemiology, Harvard T.H. Chan School of Public Health, Boston, MA, 02115, United States; Department of Population Medicine, Harvard Pilgrim Health Care Institute, Boston, MA, 02115, United States; Department of Population Medicine, Department of Population Medicine, Harvard Medical School, Boston, MA, 02115, United States; Division of Adolescent/Young Adult Medicine, Boston Children’s Hospital, Boston, MA, 02115, United States; Channing Division of Network Medicine, Harvard Medical School and Brigham and Women’s Hospital, Boston, MA, 02115, United States

**Keywords:** female sexual dysfunction, sexual satisfaction, sexual activity, sexual orientation, older adulthood

Female sexual dysfunction (FSD) is a catch-all term describing “disorders of desire, arousal, lubrication, orgasm, satisfaction, and sexual pain” among individuals assigned female at birth and is defined in part by reported distress.^1^ FSD is common and can profoundly inhibit sexual pleasure and quality of life. Sexual minority women—defined by their sexual identities, attractions, and partnerships—experience disparities compared with heterosexual women in a variety of health outcomes, including sexual health.^2^ Despite the adverse impacts of FSD, little is known about potential sexual orientation-related disparities. We used data from the Nurses’ Health Study II (NHSII) cohort to fill these gaps. We aimed to describe disparities in FSD across sexual orientation.

Nurses’ Health Study II is a longitudinal cohort established in 1989, comprising 116 429 registered nurses who reported being female at baseline; participants reside across the United States and complete biennial questionnaires. Using data from 2017—selected because this questionnaire included the most detailed multidimensional assessment available in NHSII and prior work in this cohort suggests that sexual orientation is relatively stable across midlife and older adulthood—we created a multi-dimensional 5-category measure of sexual orientation: (1) completely heterosexual (ie, no reported same-sex attractions or same-sex sexual contact or prior sexual minority identity), (2) heterosexual with past same-sex attraction, past same-sex sexual contact, or past sexual minority identity, (3) mostly heterosexual, (4) bisexual, and (5) lesbian (ie, mostly or completely homosexual). When 2017 sexual orientation data were missing, we carried forward participants’ most recently reported sexual orientation identity from 2009 or 1995.

Sexual activity and FSD were measured in 2013 using the Female Sexual Function Index-6 (FSFI-6), an instrument developed in 2010 with high sensitivity and specificity (>94%) in identifying cases of FSD.^1^ Respondents who indicated sexual inactivity for any of the 3 pertinent FSFI-6 domains (ie, arousal, lubrication, and orgasm) were coded as sexually inactive. Prior research established an FSFI-6 score of ≤19 as the cutoff for clinically-relevant FSD.^1^ Because standard FSFI-6 scoring treats sexual inactivity and no attempts at vaginal penetration as dysfunction (score of zero), we excluded participants reporting no sexual activity or no vaginal penetration from FSD analyses. Finally, individual FSFI-6 domain scores were also evaluated, with 5-point Likert scale responses categorized as low (1-2), moderate (3), and high (4-5) function.

Missingness of FSFI-6 data was high across all sexual orientation subgroups (range: 13.1%-21.4%). To avoid bias introduced by complete-case analysis, we performed multiple imputation by chained equations with 10 imputed datasets combined via Rubin’s rules to impute our primary outcome measures—sexual activity, FSFI-6 score, and FSFI-6 domain scores—using the *amelia* package in R. The imputation model included all study variables plus auxiliary variables (age, race/ethnicity, state of residence, sexual orientation in 1995 and 2009, marital status, and depressive symptoms in 2013) hypothesized to relate to missingness. Results using a complete-case approach are available in Tables S3–S5. Sample sizes for analyses using imputed data are presented as a range.

After excluding participants who had withdrawn (*n* = 22), did not respond to the 2013 questionnaire (*n* = 29 112), or had missing sexual orientation data (*n* = 2506), our analytic sample comprised 84 790 participants. For our main analyses, we used log-binomial models to estimate prevalence ratios (PRs) and corresponding 95% confidence intervals (95% CIs) for the association between sexual orientation and past-month sexual activity and FSD. Additionally, we used multinomial regression to estimate PRs and 95% CIs for the association between sexual orientation and self-reported function on each individual domain. In sensitivity analyses, we repeated complete-case analyses using the main sexual orientation schema and sexual orientation as prospectively assessed in 2009. All statistical analyses were performed in R v4.2.0.

Among 84 790 eligible participants, 76 989 (90.8%) were completely heterosexual; 4844 (5.7%) were heterosexual with same-sex attractions, partners, or prior sexual minority identity; 1770 (2.1%) were mostly heterosexual; 328 (0.4%) were bisexual; and 859 (1.0%) were lesbian. Participants were majority White (94.4%) and non-Hispanic/Latine (98.3%). In 2013, participants were aged 48-69 years and 86.2% were postmenopausal. Descriptive statistics of past-month sexual activity, continuous FSFI-6 score, and FSD status across sexual orientations are available in Table S1.

Compared with completely heterosexual participants, bisexual (PR: 0.89, 95% CI, 0.81-0.97) and lesbian (PR: 0.76, 95% CI, 0.71-0.82) participants had a lower prevalence of past-month sexual activity (Table S2). Further, sexual minority participants were more likely than completely heterosexual participants to report low satisfaction with their overall sexual life: participants who were heterosexual with same-sex partners, attractions, or prior sexual minority identity (PR: 1.11, 95% CI, 1.03-1.20), mostly heterosexual (PR: 1.34, 95% CI, 1.19-1.50), bisexual (PR: 1.17, 95% CI, 0.88-1.54), or lesbian (PR: 1.32, 95% CI, 1.12-1.56). However, bisexual and lesbian participants also had a lower prevalence of FSD compared with completely heterosexual participants (PR: 0.81, 0.65-1.01; PR: 0.87, 95% CI, 0.74-1.01, respectively). Lastly, among sexually active participants who reported attempting vaginal penetration, bisexual (PR: 1.39, 95% CI, 0.83-2.33) and lesbian (PR: 1.23, 95% CI, 0.87-1.73) participants were more likely than completely heterosexual participants to report high pain frequency. Results did not meaningfully differ in sensitivity analyses using a complete case approach (Tables S3–S5).

In this study, we found evidence that bisexual and lesbian individuals have a lower prevalence of FSD than completely heterosexual individuals. However, pervasive disparities in sexual satisfaction appeared across all sexual minority groups. Lesbian and bisexual individuals were less likely to report sexual activity and more likely to report high pain during vaginal penetration, compared with completely heterosexual participants.

Our findings are aligned with some prior research on sexual function among sexual minority populations. Decreased sexual activity and increased orgasm frequency among lesbian women compared to heterosexual women is well documented in the literature.^3^ Further, a higher prevalence of pain during vaginal penetration among bisexual and lesbian participants relative to completely heterosexual participants in our sample reflects previous research.^4^

This study suggests that both sexual orientation partnership and identity play a role in disparities in FSD prevalence, highlighting the need for multidimensional measures of sexual orientation in both survey and clinical (eg, electronic medical record) data. Additionally, our results suggest value in querying patients about specific domains of sexual function with cultural competence, including satisfaction and pain. The FSFI-6 may not achieve this degree of cultural competence, limiting the utility of our findings. Qualitative studies are needed to understand how sexual minority women define “sexual satisfaction” and to guide interventions.

Importantly, our large sample size enabled the disaggregation of multiple sexual minority subgroups, including groups that are often erased in the literature (eg, mostly heterosexual). However, given the racial, ethnic, occupational, and national homogeneity of our cohort, future work should replicate results in samples representative of the overall female sexual minority population, particularly among those who are racially/ethnically marginalized, occupationally marginalized, and/or non-Western.

Overall, sexual minority participants were less likely than completely heterosexual participants to report high sexual satisfaction. Bisexual and lesbian participants were also less likely to be sexually active in the past month. Yet among those with past-month sexual activity, bisexual and lesbian participants were less likely to meet criteria for FSD than completely heterosexual participants, despite being more likely to report pain with vaginal penetration. Further research on what shapes sexual activity and satisfaction among sexual minority women in older adulthood is needed to improve outcomes in this group and to identify protective factors against FSD across sexual orientations.

## Supplementary Material

Supplementary_material_qdag266
