# Supplementary material for: Sexual orientation differences in female sexual dysfunction in a large cohort of nurses in older adulthood
Source: J Sex Med. 2026 Aug 27;23(9):qdag266. doi: 10.1093/jsxmed/qdag266 (PMC13521505; doi:10.1093/jsxmed/qdag266)
Supplement: Supplementary file 1 [file supplementary_material_qdag266.zip › Backmatter-JSM-25-1215.R1.docx]

**Acknowledgements:** The authors would like to acknowledge the support of Pi-I ‘Debby’ Lin in the programming and manuscript quality assurance review process.

**Disclosure of Potential Conflicts of Interest:**

The authors have no conflicts to disclose.

**Authors’ contributions:**

**Tabor Hoatson:** Conceptualization, Formal analysis, Data curation, Methodology, Writing – original draft, Writing – review & editing, Visualization. **Julia C. Bond:** Conceptualization, Methodology, Writing – review & editing. **Colleen A. Reynolds:** Conceptualization, Formal analysis, Methodology, Writing – review & editing**. Julia L. Marcus:** Methodology, Writing – review & editing**. Brittany M. Charlton:** Conceptualization, Methodology, Resources, Writing – original draft, Writing – review & editing, Supervision, Funding acquisition.
