# Supplementary material for: Sexual orientation differences in female sexual dysfunction in a large cohort of nurses in older adulthood
Source: J Sex Med. 2026 Aug 27;23(9):qdag266. doi: 10.1093/jsxmed/qdag266 (PMC13521505; doi:10.1093/jsxmed/qdag266)
Supplement: Supplementary file 1 [file supplementary_material_qdag266.zip › function_executivesummary_20260714_clean.doc]

Background

- Female sexual dysfunction (FSD) can profoundly inhibit quality of life, but little is known about disparities across sexual orientation groups.

Objective

- We assessed sexual orientation-related disparities in FSD in a large cohort of female nurses in older adulthood.

Methods

- This analysis included female nurses (n=84,790) from the Nurses’ Health Study II, aged 48–69 years at the time of sexual function ascertainment in 2013.
- Sexual activity and FSD were measured using the Female Sexual Function Index-6 (FSFI-6).
- Sexual orientation was measured using five multi-dimensional categories: 1) completely heterosexual (i.e., no reported same-sex attractions or same-sex sexual contact or prior sexual minority identity (reference group), 2) heterosexual with past same-sex attraction, past same-sex sexual contact, or past sexual minority identity, 3) mostly heterosexual, 4) bisexual, and 5) lesbian (i.e., mostly or completely homosexual).
- In statistical analyses, we generated prevalence ratios (PR) and 95% confidence intervals (CI) with log-binomial and multinomial models.

Results

- Compared with completely heterosexual participants, bisexual (PR:0.89, 95%CI:0.81–0.97) and lesbian (PR:0.76, 95%CI:0.72–0.82) participants had a lower prevalence of past-month sexual activity.
- The prevalence of FSD was also lower among bisexual (PR:0.81, 95%CI 0.65–1.01) and lesbian (PR:0.87, 95%CI:0.74–1.01) participants.
- The prevalence of low sexual satisfaction was greater across all sexual minority subgroups.

Clinical Impact

- Overall, sexual minority individuals were less satisfied with their sexual lives than completely heterosexual individuals; resources to support clinicians in culturally competent discussions of sexual satisfaction with sexual minority patients are needed.

Notable Comments

- Major strengths included a large sample size, granular sexual orientation data, and psychometrically validated questionnaires.
- Limitations were misaligned timing of exposure and outcome ascertainment, requirement of past-month sexual activity and vaginal penetration to score the FSFI-6, and the racial, ethnic, occupational, and national homogeneity of the sample.

Conclusions

- To improve sexual function outcomes, further investigation is needed to identify what drives sexual activity and satisfaction among female sexual minority individuals in older adulthood, as well as identify protective factors against FSD for people of all sexual orientations.
