# Supplementary material for: Sexual orientation differences in female sexual dysfunction in a large cohort of nurses in older adulthood
Source: J Sex Med. 2026 Aug 27;23(9):qdag266. doi: 10.1093/jsxmed/qdag266 (PMC13521505; doi:10.1093/jsxmed/qdag266)
Supplement: Supplementary file 1 [file supplementary_material_qdag266.zip › supplement_survey_20260714_clean.doc]

| Supplemental Table 1. Sexual activity and prevalence of female sexual dysfunction (FSD) among participants in the Nurses’ Health Study II (N=84,790) across sexual orientation. | | | | | |
| --- | --- | --- | --- | --- | --- |
|  | Completely Heterosexual | Heterosexual with past same-sex attractions/  partners/sexual minority identity | Mostly Heterosexual | Bisexual | Lesbian |
|  | (n=76,989, 90.8%) | (n=4,844, 5.7%) | (n=1,770, 2.1%) | (n=328, 0.4%) | (n=859, 1.0%) |
| Past month sexual activity* (n, %) |  |  |  |  |  |
| Yes | 45,204 (58.7%) | 3,010 (62.1%) | 1,110 (62.7%) | 174 (53.1%) | 402 (46.8%) |
| No | 19,510 (25.3%) | 1,342 (27.7%) | 502 (28.4%) | 109 (33.2%) | 371 (43.2%) |
| Missing | 12,275 (15.9%) | 492 (10.2%) | 158 (8.9%) | 45 (13.7%) | 86 (10.0%) |
| Sexual interest, n(%) |  |  |  |  |  |
| Low interest | 44,563 (57.9%) | 3,009 (62.1%) | 1,120 (63.3%) | 207 (63.1%) | 589 (68.6%) |
| Moderate interest | 17,435 (22.6%) | 1,109 (22.9%) | 409 (23.1%) | 63 (19.2%) | 158 (18.4%) |
| High interest | 3,870 (5.0%) | 292 (6.0%) | 113 (6.4%) | 15 (4.6%) | 36 (4.2%) |
| Missing | 11,121 (14.4%) | 434 (9.0%) | 128 (7.2%) | 43 (13.1%) | 76 (8.8%) |
| Satisfaction with sexual life, n(%) |  |  |  |  |  |
| Low satisfaction | 18,513 (24.0%) | 1,349 (27.8%) | 575 (32.5%) | 90 (27.4%) | 252 (29.3%) |
| Moderate satisfaction | 15,908 (20.7%) | 1,048 (21.6%) | 392 (22.1%) | 73 (22.3%) | 202 (23.5%) |
| High satisfaction | 28,061 (36.4%) | 1,804 (37.2%) | 622 (35.1%) | 109 (33.2%) | 284 (33.1%) |
| Missing | 14,507 (18.8%) | 643 (13.3%) | 181 (10.2%) | 56 (17.1%) | 121 (14.1%) |
| Arousal during sex, n(%) |  |  |  |  |  |
| Low arousal | 19,486 (25.3%) | 1,376 (28.4%) | 480 (27.1%) | 80 (24.4%) | 206 (24.0%) |
| Moderate arousal | 17,367 (22.6%) | 1,092 (22.5%) | 398 (22.5%) | 69 (21.0%) | 150 (17.5%) |
| High arousal | 12,303 (16.0%) | 828 (17.1%) | 313 (17.7%) | 47 (14.3%) | 113 (13.2%) |
| No sexual activity | 16,385 (21.3%) | 1,101 (22.7%) | 441 (24.9%) | 88 (26.8%) | 311 (36.2%) |
| Missing | 11,448 (14.9%) | 447 (9.2%) | 138 (7.8%) | 44 (13.4%) | 79 (9.2%) |
| Lubrication during sex, n(%) |  |  |  |  |  |
| Low lubrication | 18,511 (24.0%) | 1,265 (26.1%) | 464 (26.2%) | 69 (21.0%) | 164 (19.1%) |
| Moderate lubrication | 6,944 (9.0%) | 427 (8.8%) | 182 (10.3%) | 25 (7.6%) | 47 (5.5%) |
| High lubrication | 21,067 (27.4%) | 1,400 (28.9%) | 502 (28.4%) | 89 (27.1%) | 215 (25.0%) |
| No sexual activity | 18,561 (24.1%) | 1,276 (26.3%) | 472 (26.7%) | 101 (30.8%) | 347 (40.4%) |
| Missing | 11,906 (15.5%) | 476 (9.8%) | 150 (8.5%) | 44 (13.4%) | 86 (10.0%) |
| Orgasm during sex, n(%) |  |  |  |  |  |
| Low orgasm frequency | 13,115 (17.0%) | 914 (18.9%) | 298 (16.8%) | 40 (12.2%) | 90 (10.5%) |
| Moderate orgasm frequency | 6,235 (8.1%) | 388 (8.0%) | 143 (8.1%) | 17 (5.2%) | 28 (3.3%) |
| High orgasm frequency | 27,642 (35.9%) | 1,835 (37.9%) | 744 (42.0%) | 131 (39.9%) | 319 (37.1%) |
| No sexual activity | 18,149 (23.6%) | 1,241 (25.6%) | 444 (25.1%) | 96 (29.3%) | 334 (38.9%) |
| Missing | 11,848 (15.4%) | 466 (9.6%) | 141 (8.0%) | 44 (13.4%) | 88 (10.2%) |
| Pain during vaginal penetration, n(%) |  |  |  |  |  |
| High pain frequency | 7,385 (9.6%) | 528 (10.9%) | 174 (9.8%) | 14 (4.3%) | 34 (4.0%) |
| Moderate pain frequency | 5,841 (7.6%) | 353 (7.3%) | 119 (6.7%) | 13 (4.0%) | 26 (3.0%) |
| Low pain frequency | 30,587 (39.7%) | 1,934 (39.9%) | 699 (39.5%) | 109 (33.2%) | 215 (25.0%) |
| Did not attempt intercourse | 20,690 (26.9%) | 1,520 (31.4%) | 635 (35.9%) | 146 (44.5%) | 490 (57.0%) |
| Missing | 12,486 (16.2%) | 509 (10.5%) | 143 (8.1%) | 46 (14.0%) | 94 (10.9%) |
| Among the sexually active |  |  |  |  |  |
| Continuous FSFI-6 score* (mean, SD) | 19.5 (5.5) | 19.3 (5.6) | 19.4 (5.5) | 20.5 (5.1) | 20.6 (5.1) |
| FSD** (n, %) | 18,520 (45.6%) | 1,212 (46.3%) | 430 (46.8%) | 42 (34.7%) | 90 (37.8%) |
| Missing FSFI-6 score (n, %) | 16,496 (21.4%) | 761 (15.7%) | 231 (13.1%) | 65 (19.8%) | 148 (17.2%) |
| *Calculated among those not missing an FSFI-6 score.  **FSFI-6 score ranges from 6 to 30 with lower scores representing worse sexual function.  ***FSD was defined as reporting a score of 19 or lower on the FSFI-6. | | | | | |

| Supplemental Table 2. Prevalence ratios of sexual activity and FSD* among participants in the Nurses’ Health Study II across sexual orientation. | | | | | | | | | | | | |
| --- | --- | --- | --- | --- | --- | --- | --- | --- | --- | --- | --- | --- |
|  |  | Completely Heterosexual | | Heterosexual with past same-sex attractions/  partners/sexual minority identity | | Mostly Heterosexual | | Bisexual | | Lesbian | |  |
|  |  | (n=76,989, 90.8%) | | (n=4,844, 5.7%) | | (n=1,770, 2.1%) | | (n=328, 0.4%) | | (n=859, 1.0%) | |  |
|  | Past-month sexual activity | Ref. | | 1.00 (0.98–1.02) | | 1.00 (0.97–1.03) | | 0.89 (0.81–0.97) | | 0.76 (0.71–0.82) | |  |
|  | FSD* | Ref. | | 1.01 (0.97–1.05) | | 1.01 (0.95–1.08) | | 0.81 (0.65–1.01) | | 0.87 (0.74–1.01) | |  |
|  | *FSD was defined as reporting a score of 19 or lower on the FSFI-6. | | | | | | | | | | |  |
| Supplemental Table 3. Distribution of demographic characteristics among participants in the Nurses’ Health Study II across sexual orientation using complete cases only (N=67,089). | | | | | | | | | | | | |
|  | | | Completely Heterosexual | | Heterosexual with past same-sex attractions/  partners/sexual minority identity | | Mostly Heterosexual | | Bisexual | | Lesbian | |
|  | | | (n=60,493, 90.2%) | | (n=4,083, 6.1%) | | (n=1,539, 2.3%) | | (n=263, 0.4%) | | (n=711, 1.1%) | |
| Age in years in 2013 (mean, SD) | | | 58.5 (4.6) | | 58.8 (4.5) | | 58.3 (4.6) | | 59.0 (4.8) | | 59.1 (4.5) | |
| Lifetime sexual partners, n(%) | | |  | |  | |  | |  | |  | |
| Exclusively male | | | 50,150 (82.9%) | | 3,576 (87.6%) | | 1,091 (70.9%) | | 38 (14.4%) | | 14 (2.0%) | |
| Both male and female | | | 0 (0.0%) | | 367 (9.0%) | | 433 (28.1%) | | 186 (70.7%) | | 378 (53.2%) | |
| Exclusively female | | | 0 (0.0%) | | 86 (2.1%) | | 2 (0.1%) | | 11 (4.2%) | | 206 (29.0%) | |
| No sexual partners | | | 304 (0.5%) | | 28 (0.7%) | | 9 (0.6%) | | 1 (0.4%) | | 5 (0.7%) | |
| Missing | | | 10,039 (16.6%) | | 26 (0.6%) | | 4 (0.3%) | | 27 (10.3%) | | 108 (15.2%) | |
| Race, n(%) | | |  | |  | |  | |  | |  | |
| American Indian or Alaska Native | | | 41 (0.1%) | | 2 (0.0%) | | 1 (0.1%) | | 0 (0.0%) | | 1 (0.1%) | |
| Asian or Pacific Islander | | | 690 (1.1%) | | 116 (2.8%) | | 15 (1.0%) | | 3 (1.1%) | | 8 (1.1%) | |
| Black | | | 685 (1.1%) | | 81 (2.0%) | | 14 (0.9%) | | 2 (0.8%) | | 8 (1.1%) | |
| Multiracial | | | 1034 (1.7%) | | 85 (2.1%) | | 36 (2.3%) | | 10 (3.8%) | | 13 (1.8%) | |
| White | | | 57,545 (95.1%) | | 3,750 (91.8%) | | 1,463 (95.1%) | | 244 (92.8%) | | 675 (94.9%) | |
| Other or unknown | | | 498 (0.8%) | | 49 (1.2%) | | 10 (0.6%) | | 4 (1.5%) | | 6 (0.8%) | |
| Ethnicity, n(%) | | |  | |  | |  | |  | |  | |
| Not Hispanic/Latine | | | 59,554 (98.4%) | | 3,973 (97.3%) | | 1,497 (97.3%) | | 255 (97.0%) | | 699 (98.3%) | |
| Hispanic/Latine | | | 939 (1.6%) | | 110 (2.7%) | | 42 (2.7%) | | 8 (3.0%) | | 12 (1.7%) | |
| Menopause status, n(%) | | |  | |  | |  | |  | |  | |
| Pre | | | 8,884 (14.7%) | | 508 (12.4%) | | 232 (15.1%) | | 31 (11.8%) | | 64 (9.0%) | |
| Post | | | 51,609 (85.3%) | | 3,575 (87.6%) | | 1,307 (84.9%) | | 232 (88.2%) | | 647 (91.0%) | |
|  | | | | | | | | | | | | |

| Supplemental Table 4. Sexual activity and prevalence of female sexual dysfunction (FSD) among participants in the Nurses’ Health Study II across sexual orientation using complete cases only (N=67,089). | | | | | |
| --- | --- | --- | --- | --- | --- |
|  | Completely Heterosexual | Heterosexual with past same-sex attractions/  partners/sexual minority identity | Mostly Heterosexual | Bisexual | Lesbian |
|  | (n=60,493, 90.2%) | (n=4,083, 6.1%) | (n=1,539, 2.3%) | (n=263, 0.4%) | (n=711, 1.1%) |
| Past month sexual activity (n, %) | 44,346 (73.3%) | 2,948 (72.2%) | 1,098 (71.3%) | 170 (64.6%) | 393 (55.3%) |
| Sexual interest, n(%) |  |  |  |  |  |
| Low interest | 40,226 (66.5%) | 2,745 (67.2%) | 1,040 (67.6%) | 188 (71.5%) | 527 (74.1%) |
| Moderate interest | 16,543 (27.3%) | 1,056 (25.9%) | 392 (25.5%) | 60 (22.8%) | 149 (21.0%) |
| High interest | 3,724 (6.2%) | 282 (6.9%) | 107 (7.0%) | 15 (5.7%) | 35 (4.9%) |
| Satisfaction with sexual life, n(%) |  |  |  |  |  |
| Low satisfaction | 17,861 (29.5%) | 1,305 (32.0%) | 552 (35.9%) | 87 (33.1%) | 245 (34.5%) |
| Moderate satisfaction | 15,418 (25.5%) | 1,019 (25.0%) | 385 (25.0%) | 70 (26.6%) | 193 (27.1%) |
| High satisfaction | 27,214 (45.0%) | 1,759 (43.1%) | 602 (39.1%) | 106 (40.3%) | 273 (38.4%) |
| Among the sexually active |  |  |  |  |  |
| Arousal during sex, n(%) |  |  |  |  |  |
| Low arousal | 15,805 (35.6%) | 1,114 (37.8%) | 411 (37.4%) | 56 (32.9%) | 143 (36.4%) |
| Moderate arousal | 16,606 (37.4%) | 1,031 (35.0%) | 383 (34.9%) | 68 (40.0%) | 140 (35.6%) |
| High arousal | 11,935 (26.9%) | 803 (27.2%) | 304 (27.7%) | 46 (27.1%) | 110 (28.0%) |
| Lubrication during sex, n(%) |  |  |  |  |  |
| Low lubrication | 17,199 (38.8%) | 1,183 (40.1%) | 429 (39.1%) | 61 (35.9%) | 142 (36.1%) |
| Moderate lubrication | 6,685 (15.1%) | 410 (13.9%) | 178 (16.2%) | 25 (14.7%) | 45 (11.5%) |
| High lubrication | 20,462 (46.1%) | 1,355 (46.0%) | 491 (44.7%) | 84 (49.4%) | 206 (52.4%) |
| Orgasm during sex, n(%) |  |  |  |  |  |
| Low orgasm frequency | 12,346 (27.8%) | 859 (29.1%) | 281 (25.6%) | 39 (22.9%) | 81 (20.6%) |
| Moderate orgasm frequency | 5,892 (13.3%) | 364 (12.3%) | 135 (12.3%) | 14 (8.2%) | 21 (5.3%) |
| High orgasm frequency | 26,108 (58.9%) | 1,725 (58.5%) | 682 (62.1%) | 117 (68.8%) | 291 (74.0%) |
| Pain during vaginal penetration, n(%) |  |  |  |  |  |
| High pain frequency | 6,729 (15.2%) | 476 (16.1%) | 158 (14.4%) | 12 (7.1%) | 29 (7.4%) |
| Moderate pain frequency | 5,428 (12.2%) | 335 (11.4%) | 110 (10.0%) | 13 (7.6%) | 21 (5.3%) |
| Low pain frequency | 28,415 (64.1%) | 1,806 (61.3%) | 650 (59.2%) | 96 (56.5%) | 188 (47.8%) |
| Did not attempt intercourse | 3,774 (8.5%) | 331 (11.2%) | 180 (16.4%) | 49 (28.8%) | 155 (39.4%) |
| Continuous FSFI-6 score* (mean, SD) | 19.1 (5.6) | 18.9 (5.7) | 19.0 (5.5) | 19.6 (5.2) | 19.8 (5.2) |
| FSD** (n, %) | 21,320 (48.1%) | 1,446 (49.1%) | 548 (49.9%) | 72 (42.4%) | 175 (44.5%) |
| *FSFI-6 score ranges from 6 to 30 with lower scores representing worse sexual function.  **FSD was defined as reporting a score of 19 or lower on the FSFI-6. | | | | | |

| Supplemental Table 5. Prevalence ratios of sexual activity and FSD among participants in the Nurses’ Health Study II across sexual orientation using complete cases only. | | | | | |
| --- | --- | --- | --- | --- | --- |
|  | Completely Heterosexual | Heterosexual with past same-sex attractions/  partners/sexual minority identity | Mostly Heterosexual | Bisexual | Lesbian |
|  | (n=60,493, 90.2%) | (n=4,083, 6.1%) | (n=1,539, 2.3%) | (n=263, 0.4%) | (n=711, 1.1%) |
| Past-month sexual activity | Ref. | 0.98 (0.97–1.00) | 0.97 (0.94–1.00) | 0.88 (0.80–0.96) | 0.75 (0.70–0.80) |
| FSD | Ref. | 1.02 (0.98–1.06) | 1.04 (0.98–1.10) | 0.88 (0.73–1.04) | 0.93 (0.82–1.03) |
|  | | | | | |
